# Can the development of cooking skills influence nutritional status and diet? A systematic review

**DOI:** 10.1371/journal.pone.0355940

**Published:** 2026-08-19

**Authors:** Eva Débora de Oliveira Andrade, Érika Paula Silva Freitas, Daniele de Souza Marinho do Nascimento, Rafaela Nayara da Costa Pelonha, Liana Letícia Paulino Galvão, Annamaria Barbosa do Nascimento, Grasiela Piuvezam, Manuela Mika Jomori, Thais Souza Passos, Bruna Leal Lima Maciel

**Affiliations:** 1 Graduate Program in Health Sciences, Center for Health Sciences, Federal University of Rio Grande do Norte, Natal, Brazil; 2 Graduate Program in Nutrition, Center for Health Sciences, Federal University of Rio Grande do Norte, Natal, Brazil; 3 Public Health Graduate Program, Center for Health Sciences, Federal University of Rio Grande do Norte, Natal, Brazil; 4 Department of Public Health, Center for Health Sciences, Federal University of Rio Grande do Norte, Natal, Brazil; 5 Department of Nutrition, Federal University of Santa Caratarina, Florianopolis, Brazil; 6 Department of Nutrition, Center for Health Sciences, Federal University of Rio Grande do Norte, Natal, Rio Grande do Norte, Brazil; Lusofona University of Humanities and Technologies: Universidade Lusofona de Humanidades e Tecnologias, PORTUGAL

## Abstract

Cooking skills are defined as the individual knowledge, confidence, and attitude necessary to perform culinary tasks.. Despite the growing number of primary studies investigating culinary skills and dietary outcomes, there remains a significant gap in systematic reviews synthesizing the evidence regarding their impact on the nutritional status and diet of healthy adults. The aim of this systematic review was to assess whether the development of culinary skills influences nutritional status and diet. A systematic search of studies was conducted in PubMed, Embase, Scopus, Web of Science, and SciELO. The methodological quality of the studies was assessed using the Cochrane Risk of Bias in Non-randomized Studies of Interventions (ROBINS-I) tool for non-randomized intervention studies and the Cochrane Risk of Bias for Randomized Studies (RoB 2) tool for randomized intervention studies. The Grading of Recommendations Assessment, Development, and Evaluation (GRADE) methodology was used to assess the quality of the evidence. Fifteen studies were included, most of which were classified as having a serious risk of methodological bias, such as the absence of randomization of participants and control groups. The results highlighted the importance of cooking skills and increased cooking frequency in improving diet quality among adults. However, no evidence of an association with nutritional status was found. Thus, this systematic review suggests that the development of cooking skills in healthy adults is associated with improvements in selected self-reported dietary behaviors, especially fruit and vegetable intake and cooking confidence, but the certainty of evidence is low or very low, and no reliable conclusion can be drawn regarding nutritional status. Further clinical trials are needed to strengthen the evidence base.

## Introduction

Cooking skills are understood as the set of knowledge, attitudes, and confidence required to carry out tasks related to food preparation [1,2]. These skills encompass not only the ability to cook but also competencies such as meal planning, careful food selection, mindful purchasing according to the available budget, and knowledge of techniques that minimize food waste and optimize the time dedicated to pre-preparation and meal preparation processes [3].

Evidence indicates that the development of cooking skills is strongly influenced by early exposure to culinary practices within the family environment, with greater involvement in food preparation during childhood and adolescence associated with higher skill levels in adulthood [4]. Historically, in many cultural contexts, these skills have been transmitted across generations and have often been associated with women's traditional roles in food preparation and caregiving [3,5].

Beyond their cultural dimension, cooking skills are increasingly recognized as a determinant of dietary quality. However, much of this evidence has been generated in specific population groups and contexts. In general population studies, greater proficiency and frequency of home cooking are associated with higher consumption of unprocessed and minimally processed foods, particularly fruits and vegetables [6–9]. According to the NOVA food classification system, ultra-processed foods are industrial formulations predominantly composed of substances extracted from foods and additives, with little or no whole foods remaining [10]Accordingly, higher consumption of unprocessed and minimally processed foods has been associated with higher intakes of fiber, micronutrients, and unsaturated fats [5,7,11].

In more specific contexts, such as clinical settings, cooking skills have also been identified as a relevant therapeutic and supportive strategy for improving nutritional status and quality of life, particularly among individuals with chronic noncommunicable diseases, including cancer [12–15], diabetes [16–20], cardiovascular diseases [21–24], and obesity [11,12,25]. Furthermore, evidence from targeted interventions highlights their positive influence on dietary behaviors and health outcomes in defined population groups, such as children [26–30], adolescents [31–35], and pregnant women [36–40].

Recent evidence has further reinforced the importance of dietary quality and food-related behaviors in the prevention and management of metabolic and chronic diseases. The quality of dietary fat intake has been associated with chronic disease prevention [41], while diet-derived nutrients have been linked to diabetes-related microvascular outcomes [42]. Additionally, metabolic disorders influenced by nutritional factors have been recognized as important determinants of adverse health outcomes [43]. In this context, food preparation practices and cooking skills may represent relevant upstream factors that influence dietary quality and, consequently, long-term health outcomes.

Although the body of primary research investigating culinary skills and dietary outcomes has grown in recent years, the available evidence has not yet been comprehensively synthesized in systematic reviews addressing their impact on the nutritional status and dietary patterns of healthy adults. Given that strengthening cooking skills may be a feasible strategy for health promotion and the primary prevention of chronic noncommunicable diseases, this systematic review aims to evaluate whether the development of cooking skills influences the nutritional status and diet of healthy adults.

## Materials and methods

### Protocol registration

This systematic review was conducted and reported in accordance with the Preferred Reporting Items for Systematic Reviews and Meta-Analyses (PRISMA 2020) [44] – checklist in supporting information S1 File. The review protocol was previously developed following the PRISMA-P recommendations and registered in PROSPERO (CRD42022385234), available at: https://www.crd.york.ac.uk/prospero/display_record.php?ID=CRD42022385234.

### Review question

This protocol was developed to address the following research question: Can the development of culinary skills influence nutritional status and diet in healthy adults? The question was structured according to the PECOS framework: Population (P), Exposure (E), Comparators (C), Outcomes (O), and Study Design (S), previously published in the protocol article of this review [45].

### Inclusion and exclusion criteria

This review included intervention studies published without language restrictions in scientific journals that met the eligibility criteria and involved a healthy adult population. The eligibility criteria were as follows: intervention studies that investigated cooking skills, cooking confidence, culinary and/or nutritional knowledge, nutritional status, diet quality, and dietary habits in healthy adult populations. For the purposes of this review, cooking skills interventions were defined as interventions aimed at developing at least one domain related to cooking skills, including culinary knowledge, confidence in food preparation, attitudes toward cooking, meal planning, food selection, or practical food preparation skills.

Only studies involving healthy adults were included. For this review, healthy adults were defined as individuals without diagnosed chronic diseases or clinical conditions requiring nutritional or medical treatment. Eligibility was confirmed through a full-text assessment of the studies included.

This review included both randomized controlled trials (RCTs) and quasi-experimental or non-randomized studies. The methodological distinctions among these designs were systematically considered in the assessment of risk of bias and in the stratification of studies into subgroups based on their methodological characteristics. These distinctions also informed the analytical approach adopted in synthesizing the findings. Regarding the assessment of nutritional status, data on Body Mass Index (BMI) were considered, as this parameter enabled the synthesis and comparison of outcomes across the studies included in the review.

Systematic reviews, case reports, books, conference proceedings, short communications, editorials, letters to the editor, theses, cross-sectional studies, dissertations, animal studies, and studies involving children and adolescents were excluded.

### Sources of information and literature search

The research was conducted in accordance with the protocol registered in PROSPERO to determine whether the development of cooking skills could influence the nutritional status and diet of healthy adults.

A comprehensive search strategy was developed using combinations of keywords and Boolean operators (AND and OR) in electronic databases, PubMed, Embase, Scopus, Web of Science, and SciELO, without language restrictions. The search terms were selected based on the terminology most frequently identified in studies related to cooking skills and associated constructs during the literature review and protocol development stages. MESH terms were not used in the search equations because “cooking skills,” “culinary skills”, and related terms are relatively recent in the scientific literature and do not have associated MESH terms [3,8].

The search equation was defined as: (healthy adults OR healthy people OR adults) AND (culinary skills OR cooking skills OR cooking education OR food skills OR Cooking OR Food preparation) AND (nutritional status OR body mass index OR diet OR diet quality) AND (intervention study OR clinical trial).

Additionally, a manual search was performed to include articles that may not have been retrieved from the databases listed above. The search equation for the systematic review was defined based on the established elements of three items from the PECOS strategy and aimed to maximize the sensitivity of the search. The same search equation was applied across all databases. The manual search was conducted based on relevant studies and authors previously known by the review team and considered pertinent to the review topic, resulting in the identification of two additional studies that were subsequently assessed for eligibility and included in the review. The search was conducted and finalized in May 2025.

### Study selection

**Fig 1:**
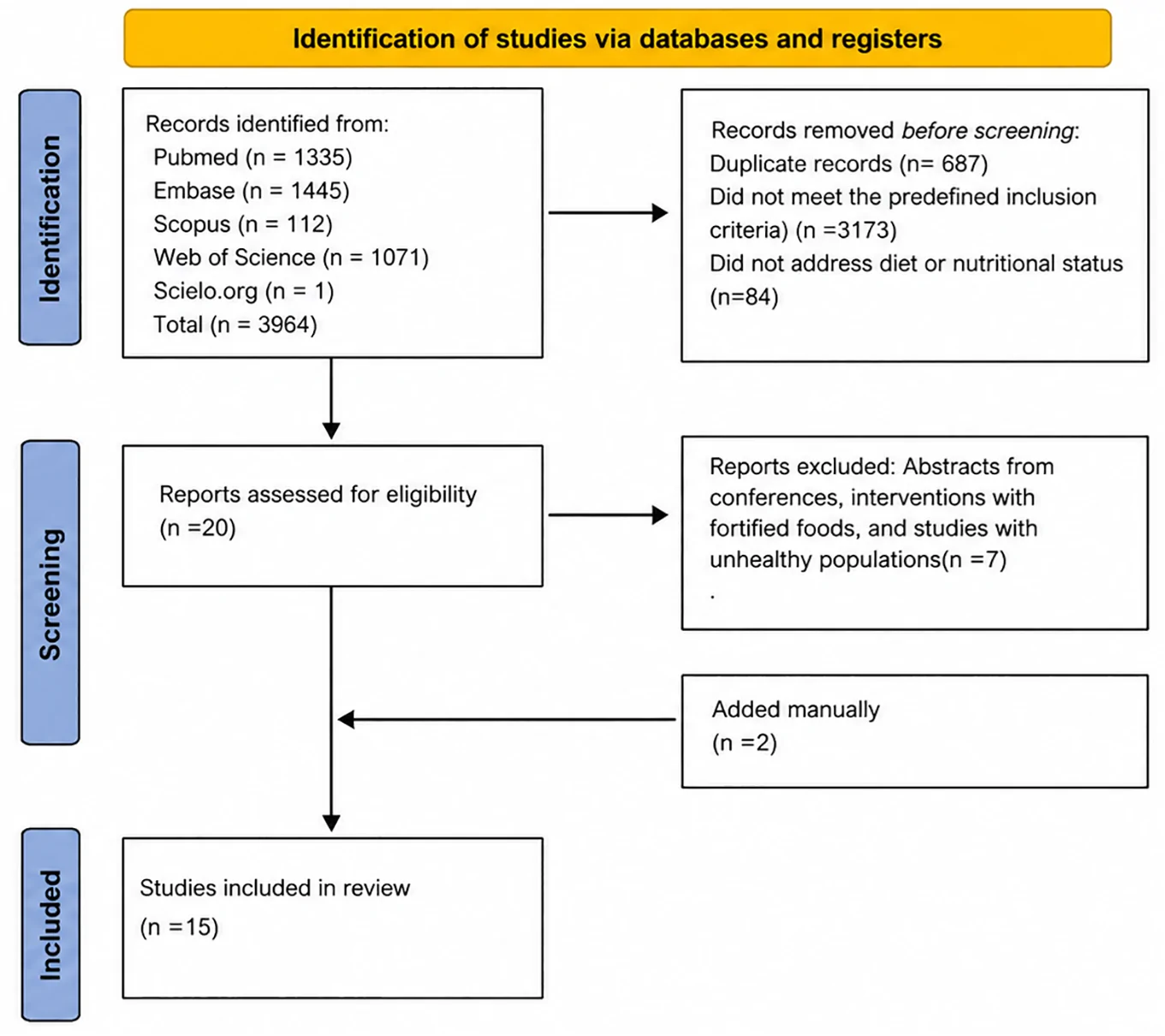
PRISMA 2020 flow diagram of the study selection process for the systematic review evaluating the influence of cooking skills development on nutritional status and diet in healthy adults. A total of 3964 records were identified through searches in PubMed, Embase, Scopus, Web of Science, and SciELO. After duplicate removal and screening procedures, 20 full-text articles were assessed for eligibility, and 15 studies were included in the final review, including two studies identified through manual searching.

The articles were uploaded to Rayyan (version 2022) [46], where duplicates were identified and removed. According to the eligibility criteria, two authors (EA and EF) performed an initial screening of the studies based on the information in their titles and abstracts. Subsequently, full-text screening was conducted by the same independent researchers Any disagreements regarding study selection were resolved through consensus meetings, with the support of the supervising researcher when necessary. A screening of the reference lists of relevant studies, as well as studies previously known by the review team, was performed to identify potentially eligible articles that had not been retrieved through the original database search. This process resulted in the identification of two additional studies that met the eligibility criteria and were included in the review.. The selection or exclusion of studies was reported using the PRISMA-P flowchart (Fig 1) in May 2025.

### Data extraction

After selecting the studies to be included in the review, two reviewers independently prepared Microsoft Excel spreadsheets containing data from these articles in May 2025. The following information was extracted and summarized: study characteristics (title, author, year, and language of publication, study location, and study design); population characteristics (health status, sample size, age, and gender of participants); methodology; description of results; relevant conclusions; and reported limitations.

### Data analysis and synthesis

For data analysis and synthesis, subgroups were defined according to the type and duration of the interventions implemented in the included studies. A narrative synthesis was conducted to summarize the findings related to nutritional status and dietary outcomes. Due to the substantial heterogeneity among studies regarding intervention characteristics, outcome assessment methods, follow-up duration, and study populations, quantitative pooling of results was considered inappropriate.

To assess the consistency of findings across studies, heterogeneity was evaluated qualitatively based on differences in study design, intervention format, outcome measures, and participant characteristics. These methodological and clinical differences were considered during the interpretation of the results and the assessment of the overall certainty of the evidence.

### Risk of bias and quality of evidence

The risk of bias was assessed by two trained independent reviewers (EA and EF) in May 2025. Non-randomized intervention studies were assessed using the Cochrane Risk Of Bias In Non-randomized Studies of Interventions (ROBINS-I) [47]. Randomized intervention studies were assessed using the Cochrane Risk of Bias tool for randomized trials (RoB 2) [48].

The Grading of Recommendations, Assessment, Development, and Evaluation (GRADE) framework was used to assess the level of certainty of the evidence. GRADE is a systematic approach for rating the certainty of evidence in systematic reviews and other evidence syntheses. GRADE classifies the certainty of evidence as high (very unlikely to change), moderate (may change), low (likely to change), or very low (very uncertain and likely to change) [49].

## Results

### Research selection

The database search conducted in PubMed, Embase, Scopus, Web of Science, and SciELO retrieved a total of 3964 records. After removal of duplicates (n = 687), 3277 records remained for screening. Of these, 3173 were excluded using Rayyan’s automated screening tools for not meeting the predefined inclusion criteria. An additional 84 records were excluded because they did not report outcomes related to diet or nutritional status.

Twenty articles were subsequently assessed for full-text eligibility, of which seven were excluded: two were conference abstracts, one focused exclusively on calcium-rich foods, and four included populations with pre-existing health conditions. As a result, 13 studies were included in the review. Two additional studies were identified through manual searching, yielding a final sample of 15 articles. The study selection process is illustrated in Fig 1.

### Characterization of the included studies

The characterization of the included studies is presented in Table 1. Of the included studies, 6 were non-randomized intervention studies, 5 were quasi-experimental studies, and 4 were randomized clinical trials, all published in English. Of the extracted studies, only 1 had a sample size greater than 1000 participants [50]. Nine studies were conducted in the United States [51–59], three studies were conducted in Australia [50,60,61], two in European countries [62,63], and one in Kenya [64]. All studies included both men and women.

**Table 1:** Characteristics of the included studies, their interventions, and impacts on nutritional status and diet.

Authors (year)	Type of study	Country	Studied population and sample size (intervention group/ control)	Duration and timing of intervention sessions	Interventions characteristics	Main results
French et al. 2024 [55]	Non-randomized intervention	United States	Residents of low-income communities 110/0	Six months, 60-minute sessions	A bilingual community intervention that combines nutritional education, culinary demonstrations, and nutritionist-led health monitoring, with pre- and post-intervention biometric assessments.	Participants showed significant improvements in clinical parameters (BMI, blood pressure, triglycerides, and, among those at higher risk, total cholesterol and LDL), as well as increased fruit intake and reduced sugar-sweetened beverage consumption.
Matias et al. 2021 [51]	Non-randomized intervention	United States	College Students 171/0	The course lasted 14 weeks and was delivered in 5 groups, with weekly sessions comprising 50 minutes of theoretical instruction and 120 minutes of practical cooking.	Educational intervention in a university setting with a teaching kitchen, combining nutritional education and hands-on culinary training to develop culinary skills and self-confidence.	The intervention improved self-efficacy, increased fruit and vegetable intake, and reduced meal skipping, with no effect on ready-to-eat or away-from-home meals.
Barr-Porter et al. 2024 [52]	Non-randomized intervention	United States	College Students 65/0	One academic semester (approximately 16 weeks), with in-person sessions of approximately 90 minutes each.	Participatory community-based intervention integrating in-person nutrition and cooking classes with text messaging to promote health and share resources related to diet, mental health, and campus services.	Intervention resulted in a reduction in days of poor mental health and anxiety, as well as an increase in vegetable intake and overall diet quality.
Ylitalo et al. 2023 [53]	Non-andomized intervention	United States	Residents of low-income communities 33/0	4 to 6 weekly sessions over two-year cycles between 2022 and 2023, 90-minute sessions.	Food as Medicine intervention in a community health center, integrating the prescription of fruits and vegetables with hands-on classes in a teaching kitchen to promote culinary skills and eating behavior.	The intervention increased culinary self-efficacy and dietary self-management, with 44% of participants reporting higher vegetable consumption.
French et al. 2024 [54]	Non-randomized intervention	United States	College Students 50/79	Duration: 1 semester (14 weeks), weekly sessions of 50 min (theory) + 120 min (culinary practice).	Educational intervention in a university food skills course with a teaching kitchen, integrating theoretical classes and culinary labs to develop meal planning, preparation of accessible meals, and culinary skills.	The course was associated with greater self-efficacy for healthy eating, increased cooking frequency, reduced meal skipping, and a smaller decline in vegetable consumption.
Fernando et al. 2024 [62]	Non-randomized intervention	Spain	Healthcare professionals 20/0	4 weeks, with four weekly sessions, each lasting 480 minutes.	Intensive Culinary Medicine program, combining nutrition education and hands-on culinary training to build culinary skills, food management, and counseling confidence.	After the program, greater adherence to the Mediterranean diet and improvements in culinary confidence and techniques were observed, highlighting the value of practical training in professional education.
Caspi et al. 2017 [56]	Quasi-experimental study	United States	Food-insecure adults 63/0	6-week program, weekly sessions between 120–150 minutes each.	Community intervention in food shelves that integrated nutritional education and practical cooking classes for food-insecure adults, with collective meal preparation and provision of ingredients for home use.	The intervention improved diet quality and cooking skills, but only cooking skills remained after 30 days.
Flego et al. 2014 [50]	Quasi-experimental study	Australia	Residents of low-income communities 1526/434	10-week program, with weekly 90-minute sessions.	Community-based cooking skills intervention, delivered through structured weekly hands-on classes focused on cooking from scratch, integrating nutrition messages, meal planning, and the promotion of healthy eating behaviors	The intervention increased cooking confidence, raised vegetable intake, and reduced the consumption of ready-made meals.
Asher et al. 2022 [60]	Quasi-experimental study	Australia	Health and education professionals 30/0	Total duration of 5 weeks, organized into 5 weekly modules, with an estimated workload of 120–180 minutes per module.	Online culinary nutrition/culinary medicine educational intervention for professionals, integrating evidence-based theoretical content with home-based culinary activities focused on vegetable preparation and food skills.	Intervention increased the consumption and variety of fruits and vegetables, cooking confidence, and nutritional knowledge.
Kearsey et al. 2023 [61]	Quasi-experimental study	Australia	Vulnerable adults at increased risk of food insecurity 258/0	Total duration of 6 weeks, consisting of weekly sessions of 150 minutes each.	Nutrition lectures and culinary practice focused on healthy choices, meal planning, and accessible recipes.	Intervention increased fruit and vegetable consumption and cooking confidence, with some effects sustained after the program.
Hutchinson et al. 2016 [63]	Quasi-experimental study	United Kingdom	Adults who frequent the Ministry of Food's center 795/0	Total duration of 8 weeks, consisting of weekly 90-minute sessions.	Community-based cooking skills intervention for adults, delivered through weekly hands-on classes integrating nutrition education, preparation of healthy and affordable meals, and the development of cooking confidence.	Intervention increased fruit and vegetable intake, reduced consumption of unhealthy snacks, and improved cooking confidence.
Merchant et al. 2023 [64]	Randomized clinical trial	Kenya	Small farmers in western Kenya 316/187	Total duration of 12 months, with sessions of approximately 90–120 minutes each.	The study (2016–2019) included agricultural modules, household nutrition education, and community-based culinary training.	The intervention increased dietary diversity and the consumption of local vegetables.
Pope et al. 2021 [57]	Randomized clinical trial	United States	College Students 43/10	The total duration of the intervention was 12 weeks, with 120-minute sessions.	An intervention for university students that combined hands-on cooking classes in a teaching kitchen with the provision of meal kits, aiming to develop culinary skills, self-efficacy, and meal planning.	The cooking classes significantly increased food agency scores and raised the frequency of meal preparation.
Kramer et al. 2023 [58]	Randomized clinical trial	United States	Adults self-identifying as Black or African American 72/68	Brief intervention, single session (15 minutes), with a 1-week follow-up.	Brief experimental intervention based on exposure to a short cooking video (versus a text-based control), followed by a reflective writing activity, aimed at influencing perceptions, attitudes, and intentions related to food preparation.	The video increased intentions to cook, which were associated with greater cooking practice and improved diet quality in the following week.
Metcalfe et al. 2022 [59]	Randomized clinical trial	United States	Residents of low-income communities 88/52	7-week program; weekly sessions of approximately 90 minutes each.	Community-based intervention with weekly nutrition education and cooking classes, focused on hands-on food preparation and healthy food choices.	The intervention group increased fruit and vegetable intake, without changes in cooking frequency, but with greater variety and self-efficacy.

The interventions included in the review exhibited substantial heterogeneity in total duration and session length. Programs of moderate duration, ranging from 5 to 14 weeks, with weekly sessions lasting between 60 and 150 minutes, were the most common, particularly in community-based and university settings [50–56,59,61,63]. Interventions conducted in academic environments frequently combined theoretical and practical components, resulting in higher weekly time commitments, such as 50 minutes of theoretical instruction combined with 120 minutes of hands-on culinary practice [51,54,55].

In contrast, community-based interventions predominantly adopted single weekly sessions lasting 90–120 minutes [50,53,56,59,62]. Short-term intensive formats were also identified, including a 4-week program with extended sessions lasting up to 480 minutes, targeted at healthcare professionals [62], as well as longer-duration interventions lasting up to 12 months, in which in-person sessions occurred periodically over time [64]. Finally, one study evaluated a brief intervention, consisting of a single session of approximately 15 minutes, followed by short-term follow-up [58], highlighting the wide variability in temporal formats across the included studies.

Among the studies included in this review, only one assessed nutritional status using body mass index (BMI). Although BMI was measured before and after the intervention, the results did not show a statistically significant impact on BMI [55] (Table 1).

Regarding the participants’ diet, an increase in the consumption of fruits, vegetables [51], and whole grains was observed [52], as well as a reduction in the intake of sugar-sweetened beverages and the frequency of skipping meals [55]. Moreover, two studies reported enhancements in overall diet quality, as evidenced by higher scores on healthy eating indices such as the HEI-2010 [52,56]. These changes were also accompanied by an increased frequency of home-cooked meals [52,56], indicating that culinary practice contributed to more structured and healthier food choices.

Another consistent finding was the improvement in cooking self-efficacy and the use of dietary self-management strategies following the interventions, promoting a more positive and conscious relationship with food [53,56,62]. Across different settings, including food-insecure populations [61], university students [60], and small-scale producers [64], participants reported greater confidence in preparing healthy meals and increased consumption of fresh and minimally processed foods.

### Quality assessment and risk of bias

The risk of bias assessment for included studies was performed using tools specific to each design (Table 2). We did not merge the risk of bias classifications from RoB 2 and ROBINS-I, as methodological literature strongly advises against this practice due to inherent differences in study types and the bias sources evaluated [47,48]. RoB 2 was applied to randomized controlled trials (RCTs), focusing on randomization-related biases, while ROBINS-I assessed non-randomized studies of interventions (NRSIs), prioritizing confounding bias [47,48]. This distinction is crucial, as even an NRSI with a low risk of bias doesn't hold the same strength of causal evidence as an RCT [47,65]. Consequently, risk of bias classifications were presented according to the instrument used.

**Table 2:** Classification of studies by risk of bias.

Authors (year)	Type of study	Risk of bias classification
French et al. 2024 [55]	Non-randomized intervention	Serious risk of bias Due to confounding arising from the non-randomized pre–post design without a control group and voluntary participation^1^
Matias et al. 2021 [51]	Non-randomized intervention	Serious risk of bias Due to confounding and participant selection arising from the uncontrolled pre–post design, convenience sampling, inclusion of nutrition students, lack of adjustment for key socioeconomic confounders^1^
Barr-Porter et al. 2024 [52]	Non-randomized intervention	Serious risk of bias Due to uncontrolled confounding from the pre–post design with self-selected participants and no control group, combined with moderate bias from missing data, self-reported outcomes, and exploratory subgroup analyses^1^
Ylitalo et al. 2023 [53]	Non-randomized intervention	Moderate risk of bias Due to substantial loss to follow-up and reliance on self-reported outcomes in a non-blinded pre–post design, without sensitivity analyses to address missing data^1^
French et al. 2024 [54]	Non-randomized intervention	Serious risk of bias Due to residual confounding from the non-randomized design, baseline differences between groups, and incomplete adjustment for key determinants of dietary behaviors^1^
Fernando et al. 2024 [62]	Non-randomized intervention	Serious risk of bias Due to uncontrolled confounding arising from the non-randomized pre–post design with self-selected participants and lack of adjustment for key baseline characteristics^1^
Caspi et al. 2017 [56]	Quasi-experimental study	Moderate risk of bias Due to incomplete outcome data and reliance on self-reported dietary intake and cooking skills in a non-blinded pre–post design, without sensitivity analyses to address missing data^1^
Flego et al. 2014 [50]	Quasi-experimental study	Moderate risk of bias Due to substantial attrition across follow-up time points and reliance on self-reported outcomes without blinding, with no sensitivity analyses to assess the impact of missing data^1^
Asher et al. 2022 [60]	Quasi-experimental study	Moderate risk of bias Due by incomplete outcome data and self-reported dietary and skill measures without blinding, increasing susceptibility to response bias^1^
Kearsey et al. 2023 [61]	Quasi-experimental study	Serious risk of bias. Due to lack of adjustment for key sociodemographic and baseline prognostic confounders in a non-controlled pre–post design^1^
Hutchinson et al. 2016 [63]	Quasi-experimental study	Moderate risk of bias Due to substantial attrition with complete-case analysis and reliance on self-reported outcomes without blinding^1^
Merchant et al. 2023 [64]	Randomized clinical trial	High risk of bias Due to the substantial loss of participants during follow-up and the insufficient information to ensure that the lack of data on outcomes did not bias the estimated effects of the intervention^2^
Pope et al. 2021 [57]	Randomized clinical trial	High risk of bias Due to the high dropout rate throughout the follow-up phases, and the consequent lack of data on the results, this is probably related to participant engagement^2^
Kramer et al. 2023 [58]	Randomized clinical trial	Some concerns Due to lack of blinding of participants and researchers, reliance on self-reported outcomes, and potential impact of missing data at follow-up, although attrition was low^2^
Metcalfe et al. 2022 [59]	Randomized clinical trial	Low risk of bias^2^

^1^Bias analyzed by risk of bias in non-randomized intervention studies (ROBINS-1)

^2^Bias analyzed by risk of bias in randomized studies (RoB 2)

Among the non-randomized studies, most were classified as presenting a serious risk of bias, primarily due to the lack of blinding of participants and researchers, the use of convenience sampling, and losses to follow-up, all of which may compromise internal validity and increase the risk of selection bias and residual confounding. Ylitalo et al. [53] was the only exception, classified as having a moderate risk of bias due to the more rigorous control of confounding variables, although limitations related to missing data were still observed.

Regarding the randomized controlled trials, two studies [57,64] were classified as having a high risk of bias, mainly due to incomplete or missing outcome data, which may substantially affect the robustness and validity of the reported findings.

For the analysis of the quality of evidence, the certainty of the findings was assessed using the GRADE method, grouped by study type and outcome, recognizing the specific characteristics of each design and the specific nature of the results for each outcome [49,65,66]. The GRADE assessment for the nutritional status outcome also resulted in very low certainty, due to high risk of bias and an extremely small sample size, severely compromising the precision and confidence in the findings (Table 2). Regarding the dietary outcome, the certainty of the evidence was very low for non-randomized and quasi-experimental studies, mainly due to the high risk of bias and the lack of comparability between groups. For randomized clinical trials, the certainty was low, despite a more favorable starting point, still affected by the high risk of bias prevalent in most studies.

### Association of nutritional status and diet with cooking skills

The summary of the association between nutritional status and diet and cooking skills is shown in Table 3.

**Table 3:** Evidence summary and quality of the evidence of the influence of cooking skills development on nutritional status and diet.

Outcome	Type of study	Results	Number of studies (participants)	Evidence summaries	Quality of the evidence*
Nutritional status	Non-randomized intervention [55]	No relationship was found between BMI and cooking knowledge and technique, and eating habits and knowledge, cooking technique and cooking skills	1 (110)	No association	⊕●●● Very low evidence: Non-randomized intervention with a serious risk of bias and an extremely small sample size
Diet	Non-randomized intervention [51–55,62]	Cooking more frequently was associated with higher vegetable and fruit consumption. Greater confidence in cooking skills was associated with higher consumption of fresh and whole foods. Sugary beverage consumption decreased after the intervention. A decrease in meal skipping was also associated with greater cooking skills	6 (528)	Positive association	⊕●●● Very low evidence: Predominance of non-randomized studies with a high risk of bias
Diet	Quasi-experimental studies [50,56,60,61,63]	Culinary interventions were associated with improved diet quality and increased consumption of vegetables and fruits. Participants reported lower consumption of unhealthy snacks and ready-to-eat ultra-processed foods	5 (3106)	Positive association	⊕●●● Very low evidence: High risk of bias and lack of comparability of the groups
Diet	Randomized clinical trials [57–59,64]	Participants in cooking classes showed higher consumption of fruits and vegetables and a higher frequency of meal preparation	4 (843)	Positive association	⊕⊕●● Low evidence: Predominant presence of high risk of bias in most studies, significantly compromised the reliability of the results

*Assessed using the **g**rading of **r**ecommendation, **a**ssessment, **d**evelopment, and **e**valuation (GRADE) framework: ⊕•••: very low grade; ⊕⊕••: low grade; ⊕⊕⊕•: moderate grade; ⊕⊕⊕⊕: high grade.

Analysis of the included studies revealed no evidence of an association between the development of culinary skills and nutritional status. The only non-randomized intervention study, conducted with 110 participants, found no statistically significant relationship between body mass index, culinary knowledge, cooking techniques, and eating habits. These findings indicate a lack of a direct association between the development of culinary skills and nutritional status. Furthermore, the evidence was classified as very low quality due to the study design, high risk of bias, and small sample size, which compromises the reliability of the results.

Regarding dietary outcomes, the studies reported more consistent findings. Non-randomized interventions, comprising six investigations [51–55,62] with 528 participants suggested that a higher frequency of meal preparation and greater confidence in cooking were associated with increased consumption of fruits [51–53], vegetables [54,62], fresh, and whole foods, as well as reductions in sugar-sweetened beverage intake and meal skipping [55].

Despite these positive findings, the overall certainty of the evidence was rated as very low, largely due to the predominance of studies at high risk of bias, including non-randomized designs with inadequate control of confounding factors [51,52,54,55,62].

Similar results were observed in five quasi-experimental studies (n = 3106), in which culinary interventions were associated with improvements in overall diet quality [56,50], increased fruit and vegetable consumption [60,61], and reduced intake of unhealthy snacks and ready-to-eat ultra-processed foods [63]. However, the certainty of the evidence from these studies was also classified as very low, given the substantial risk of bias, particularly due to insufficient control for confounding variables and limited comparability between the intervention and control groups.

Finally, four randomized controlled trials (n = 843) reported that participants who attended cooking classes demonstrated higher fruit and vegetable intake and greater frequency of home meal preparation [57–59,64]. Although these findings suggest potential benefits, the certainty of evidence was rated as low, primarily due to the high risk of bias in some trials, including incomplete control of confounding factors and missing follow-up outcome data [57,64], which limit the robustness and reliability of the conclusions

## Discussion

This systematic review aimed to critically examine the influence of developing cooking skills on nutritional status and diet in healthy adults from different populations and study designs. Overall, no evidence was found to support a direct association between culinary skills and nutritional status, as only one non-randomized study assessed BMI and found no statistically significant effects. In contrast, more consistent positive associations were observed for dietary outcomes, with interventions generally linked to increased fruit and vegetable consumption, improved overall diet quality, and more frequent home meal preparation. However, despite these favorable results, the certainty of the evidence ranged from very low to low, mainly due to the high risk of bias, non-randomized designs, incomplete control of confounding factors, and substantial heterogeneity among studies.

These findings are consistent with previous literature, which suggests that the effects of culinary interventions on BMI are neither immediate nor linear [67–69]. Nutritional status is influenced by a complex interaction of determinants that go beyond culinary skills, including physical activity, socioeconomic context, access to food, social environment, and sleep patterns [67,70]. Consequently, detecting measurable changes in anthropometric outcomes may require longer intervention durations and more robust methodological designs, particularly well-conducted randomized controlled trials with larger and more diverse samples. Such designs would increase internal validity, reduce bias, and improve the generalizability of results [68,69,71].

Furthermore, the lack of significant findings regarding BMI in this review may also be attributed to the characteristics of the included studies, which comprised healthy adult populations, a group in which substantial variations in this parameter are less expected [68]. It is also plausible that interventions exclusively delivered at the group level may not be sufficient to produce measurable changes in anthropometric outcomes. In this regard, incorporating individualized strategies alongside collective interventions could enhance effectiveness by addressing specific needs, motivations, and behavioral patterns of participants [71,72].

Moreover, the absence of associations observed in the present analysis may be related to the profile of the included population. Studies targeting individuals with overweight or obesity, combined with individualized approaches in addition to cooking skills interventions, may be more likely to demonstrate significant impacts on BMI and other anthropometric indicators [72,73].

Regarding diet, results consistently showed that interventions focused on cooking skills led to significant improvements in eating habits, underscoring the importance of these skills for promoting healthier eating. An increase in the consumption of fruits [50], vegetables [52], and whole grains was observed [56–58], as well as a reduction in the intake of sugar-sweetened beverages [55]and the frequency of meal skipping [51]. These results are consistent with the literature indicating that cooking ability is a predictor of higher-quality diets. Previous studies suggest that individuals with greater cooking skills tend to prepare more home-cooked meals, consume fewer ultra-processed foods, and have a higher intake of fruits and vegetables [4,5].

Although the certainty of the evidence remains limited, the observed improvements in dietary behaviors may be relevant from a public health perspective. Previous studies have demonstrated associations between dietary quality, metabolic health, and chronic disease prevention [41–43]. Therefore, interventions that promote healthier eating behaviors by developing cooking skills may help improve dietary patterns among healthy adults.

Despite these consistent findings, the risk of bias analysis identified significant methodological limitations in the available studies. Most non-randomized studies presented a serious risk of bias, mainly due to confounding, selection bias, and insufficient adjustment for relevant covariates. Similarly, most randomized controlled trials (RCTs) demonstrated a high risk of bias, often related to deviations from intended interventions, lack of blinding, and incomplete outcome reporting. Consequently, the certainty of the evidence, as assessed using the GRADE system, was rated as very low for non-randomized and quasi-experimental studies, and low for RCTs on dietary outcomes. For nutritional status, the certainty of evidence was also considered very low, since it relied on a single non-randomized study with a serious risk of bias and a very limited sample size [47–49,66].

This scenario underscores the urgent need for studies with more rigorous methodological designs, particularly well-conducted RCTs with representative samples and standardized intervention protocols, to strengthen the reliability and applicability of the findings [68,69,71]. However, it is important to recognize that, in dietary interventions, certain methodological limitations are inherent to the study design. Blinding, for example, is often not feasible, since participants are generally aware of the foods they consume or the behaviors they adopt, and the professionals conducting the intervention are also not blinded. This limitation has already been addressed and discussed in the literature and may increase the risk of performance and detection bias, especially in studies that rely on self-reported results [74].

Although the certainty of the evidence for dietary outcomes was rated as low or very low, mainly due to risk of bias and limited comparability between groups, the findings are consistent with cross-sectional evidence. In this context, Tani et al. (2020) demonstrated that higher cooking skills were associated with healthier food choices among adults, reinforcing the idea that the development of such skills can act as mediators of eating behavior, promoting a more mindful, positive relationship with food. The literature also reinforces that a higher frequency of home meal preparation is associated with better diet quality [68,69], supporting the notion that mastering cooking skills represents an effective strategy for the prevention and management of non-communicable chronic diseases. Accordingly, interventions aimed at strengthening these skills may be regarded as a feasible and effective public health approach to promoting healthy and adequate diets.

The main methodological limitation of this review was the high heterogeneity among the included studies, which prevented the meta-analysis. This challenge is recognized in the literature and stems primarily from the lack of standardization in the definition of interventions and the measurement of outcomes. Another limitation is that the restriction to peer-reviewed studies indexed in bibliographic databases may have excluded relevant grey literature.

Among its strengths, this review is one of the first to specifically examine the influence of cooking skills interventions on nutritional status and diet in healthy adults, thereby addressing a relevant gap in the literature. Previous systematic reviews have predominantly focused on specific populations, such as children [26–30], individuals with chronic diseases [12–24], vulnerable groups, or broader nutrition education programs [31–35] without positioning cooking skills as the primary intervention. Thus, the main contribution of the present review lies in consolidating and critically appraising the available evidence specifically for healthy adults.

Importantly, although several years have passed since Reicks et al.’s systematic review was published. [75], many of the methodological limitations previously identified persist in the current body of evidence. Like the concerns raised by Reicks et al., the literature remains characterized by small sample sizes, a predominance of non-randomized designs, short follow-up periods, reliance on self-reported dietary measures, and insufficient control for confounding factors. These recurring limitations indicate that, despite growing interest in culinary interventions, advances in methodological rigor have been limited. Unlike Reicks et al., which included both healthy adults and individuals with health conditions, the present review narrowed its focus exclusively to healthy adults, yet the same structural weaknesses in study design and reporting remain evident.

This approach enabled a broader search and the inclusion of more relevant studies. Such delimitation strengthened the robustness of the findings, provided a more consistent basis for future research, and may support the development of public policies aimed at promoting health by enhancing cooking skills among healthy adults. To further advance this field of knowledge, future investigations should prioritize high-quality randomized clinical trials conducted with representative samples and long-term follow-up to increase the reliability and applicability of the results.

## Conclusions

This systematic review indicated that the development of cooking skills in healthy adults may be associated with improvements in selected dietary behaviors, especially fruit and vegetable intake and cooking confidence, but the certainty of evidence is low or very low. Considering nutritional status, no reliable conclusion can be drawn, considering the lack of available data. The methodological heterogeneity of interventions and the low certainty of some outcomes highlight the need for more rigorous and well-designed studies. Thus, this review suggests that cooking skills interventions may contribute to promoting healthier dietary behaviors, while also underscoring the urgency of high-quality clinical trials to provide a stronger evidence base regarding their effects on diet and nutritional status.

## Supporting information

S1 File Prisma 2020 checklist file. (DOCX)
